# Esmraldi: efficient methods for the fusion of mass spectrometry and magnetic resonance images

**DOI:** 10.1186/s12859-020-03954-z

**Published:** 2021-02-08

**Authors:** Florent Grélard, David Legland, Mathieu Fanuel, Bastien Arnaud, Loïc Foucat, Hélène Rogniaux

**Affiliations:** 1 UR BIA, INRAE, 44316 Nantes, France; 2 BIBS Facility, INRAE, 44316 Nantes, France

**Keywords:** Image fusion, Image processing, Image registration, Spectra processing, Mass spectrometry imaging, Magnetic resonance imaging

## Abstract

**Background:**

Mass spectrometry imaging (MSI) is a family of acquisition techniques producing images of the distribution of molecules in a sample, without any prior tagging of the molecules. This makes it a very interesting technique for exploratory research. However, the images are difficult to analyze because the enclosed data has high dimensionality, and their content does not necessarily reflect the shape of the object of interest. Conversely, magnetic resonance imaging (MRI) scans reflect the anatomy of the tissue. MRI also provides complementary information to MSI, such as the content and distribution of water.

**Results:**

We propose a new workflow to merge the information from 2D MALDI–MSI and MRI images. Our workflow can be applied to large MSI datasets in a limited amount of time. Moreover, the workflow is fully automated and based on deterministic methods which ensures the reproducibility of the results. Our methods were evaluated and compared with state-of-the-art methods. Results show that the images are combined precisely and in a time-efficient manner.

**Conclusion:**

Our workflow reveals molecules which co-localize with water in biological images. It can be applied on any MSI and MRI datasets which satisfy a few conditions: same regions of the shape enclosed in the images and similar intensity distributions.

## Background

The identification of molecules in metabolic pathways is essential for the physiological understanding of an organism. For instance, the growth of plant tissues is the result of various metabolic reactions. These reactions involve hundreds of molecules and depend on the water activity in localized regions of the plant [1]. In particular, the variations of the water viscosity across different stages of development is often responsible for the changes in tissue morphology [2]. In the wheat grain, water has a strong impact on the developmental stages, and on the functional properties for milling and baking in the mature grain. Cell walls are believed to be key actors in water diffusion and distribution, similarly to the observations made for barley grains [3]. However, a clear correlation between cell wall structures and water distribution has never been established. We seek to identify the chemical structures of cell wall molecules which correlate spatially with the distribution of water.

This investigation can be led through image fusion, understood here as the joint analysis of images from two imaging techniques.

On one hand, Matrix assisted laser desorption ionization–mass spectrometry imaging (MALDI–MSI) is an acquisition technique which produces ion images, that is to say images of ionized molecules on the surface of a tissue. This acquisition technique is increasingly popular, with applications ranging from clinical research [4], forensics [5], to plant biology [6]. Molecules are not tagged prior to the acquisition, which makes this technique extremely relevant for exploratory research. However, the dimensionality of the image data is very high: hundreds of ions, that is to say charged molecules, are detected. Moreover, their distribution does not necessarily reflect the anatomical structures in the tissue.

On the other hand, magnetic resonance imaging (MRI) images highlight the structural organization of a tissue. The intensity in the images reflects the proton density, which is essentially correlated to the amount of water. The difference in nature between the two imaging techniques makes it difficult to analyze the images jointly. Indeed, even though the pixel resolution is similar, the embedded objects do not strictly have the same geometrical shape. In fact, MRI is performed from the whole object while MSI operates from thin sections. In addition, several steps in MSI are required for tissue preparation, which induce local tissue deformations and shrinkage. We propose a new workflow which addresses these discrepancies in order to merge the information from both images. This workflow ultimately makes it possible to identify molecules from MALDI–MS images whose distribution correlates with the distribution of water in MRI. We show one application of this workflow on wheat grain images.

Fusion approaches for MSI combine other imaging modalities to supplement the information given by MALDI–MS images. Buchberger et al. [7] describe a typical workflow for the fusion of MALDI–MS images: (1) image pre-processing, (2) segmentation, (3) co-registration, (4) joint data analysis: correlation, prediction. In the following, we describe the steps which are shared by fusion workflows, regardless of the modalities chosen in combination with MS images. The *pre-processing* step usually involves reducing the amount of data in the MS images. *Segmentation* consists in extracting the object of interest in the images from each modality. In MS images, ion images enclose different, yet complementary information. The object of interest is generally segmented by using several carefully selected ion images. *Registration* methods align images from different modalities by estimating the transformation which ensures the best matching between the images. The local deformations in MS images can be compensated by deformable registration methods, such as a grid of B-spline control points [8]. Finally, the images from both modalities are *analyzed jointly*. For instance, the molecular distribution in MS images can be mapped in higher resolution images [9], or the localization of molecules can be found in labelled anatomical regions [10].

Recent methods have been proposed to merge the information between MSI and MRI images. Verbeeck et al. [11] build a brain atlas by co-registering MSI and MRI images. First, the brain in the MS image is segmented by extracting a representative image. This image corresponds to a manually selected score image from the principal component analysis (PCA) decomposition of the MS image. Then, the representative image is registered onto the MRI image using a deformable method. Abdelmoula et al. [12] combine MRI and MSI data in order to identify the molecular distribution in different cell compartments. The shape is segmented in the MALDI–MS image by a hierarchical version of the t-SNE algorithm, which builds different levels of detail across several ion images. This segmented shape is registered onto the MRI image with a deformable registration method. Correspondences with the MRI are established visually. Both these methods use manual steps, which impedes the analysis process.

**Fig. 1:**
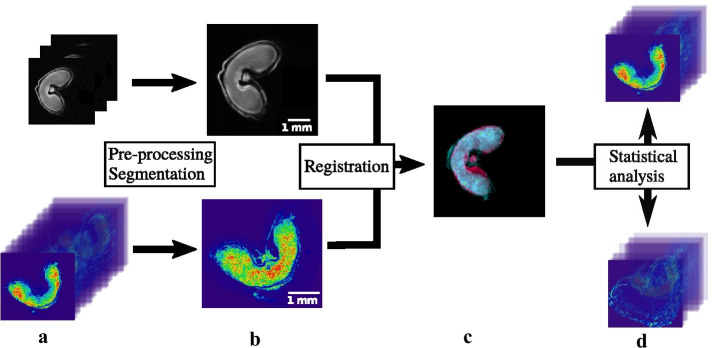
Overview of the proposed workflow. Steps for the fusion the fusion of **a** MRI (top row) and MALDI–MS (bottom row) images of a wheat grain. **b** The first step consists in extracting a representative shape through image reduction and segmentation. **c** The segmented shape in MALDI–MS is registered onto the MRI shape. This step involves linear and deformable registration methods. **d** Joint correlative analysis of the images: selection of the ion images in MALDI–MS which exhibit a similar spatial distribution as the distribution of water in MRI

**Fig. 2:**
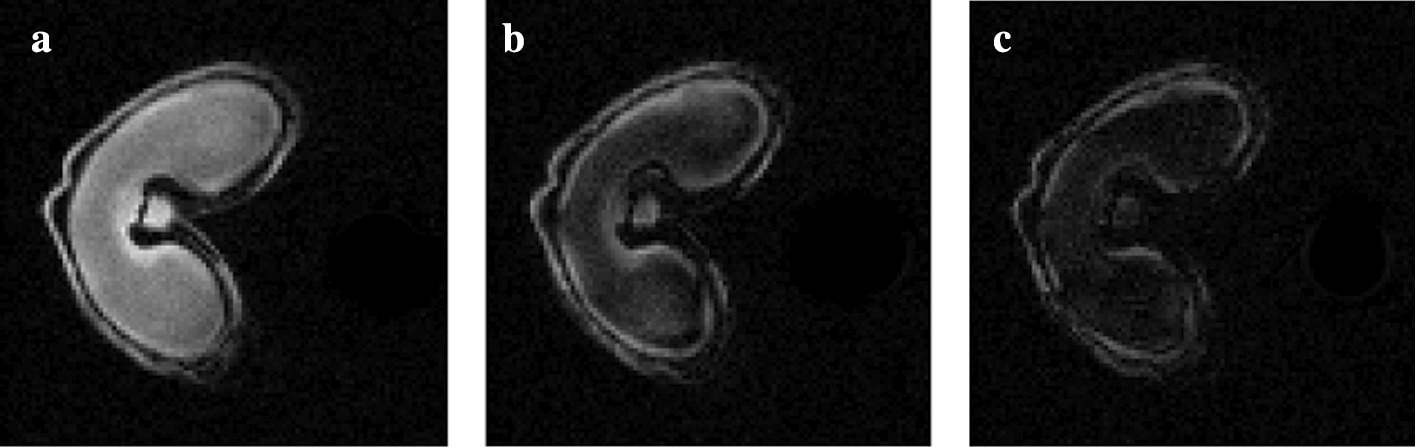
MRI images obtained at different echoes. **a** first echo, **b** second echo and **c** third echo. The intensity is decreasing over time following an exponential function

We propose a new workflow which aims at merging the information from MRI and MSI, to find molecules whose localization correlates with the distribution of water in the wheat grain (see Fig. 1). We select and propose efficient methods, which do not require manual steps. First, the grain is segmented in both images. The MRI image is denoised with an algorithm specifically suited for these images. The temporal dimension of the MRI image is reduced and a simple thresholding scheme allows to segment the grain. The MALDI–MS image is reduced in its spectral dimension by a new peak detection algorithm. Then, the grain is segmented by region growing on a subset of representative, non-noisy images. We propose a new measure to identify this subset. Secondly, the segmented shape from the MALDI–MS image is registered onto the segmented shape in the MRI image. An initial linear registration method is used, and allows for a global alignment of the shapes. However, this registration step does not compensate for the local geometrical deformations induced by the sample preparation in MALDI–MSI. Thus, the first linear registration step is followed by a deformable registration step. Thirdly, we find spatial correlations between the water distribution in MRI images and the ion images from MALDI–MSI. This is achieved by finding proximities between the MRI image and the ion images in MSI in the space of components generated by matrix factorization techniques. This workflow is validated by an extensive evaluation with comparisons to state-of-the-art methods.

## Methods

In this section, we introduce a new workflow for the fusion of 2D MALDI–MS and MRI images.

### Image acquisition

Whole wheat grains (Triticum aestivum L. cv Recital, 250 degree celsius per day after flowering) are imaged in MRI without sample preparation. Proton density images are obtained using multi-slice multi-gradient echo pulse sequence. This sequence produces images of decreasing signal intensity over several discrete time steps, called echoes (see Fig. 2). Here, eight echoes are obtained, starting at time $t=1.26~\text {ms}$ and spaced apart by one millisecond. The signal decreases over time following a negative exponential function. The resulting image is a four dimensional stack (3D + echo time) of size $100\times 100\times 14\times 8$ pixels. The transverse 2D slices have a pixel size of 50 $\mu \text {m}$, and a slice thickness of 0.5 mm. Various instrumental bias, such as magnetic field inhomogeneities, induce noisy intensities in the image with Rician distribution.

**Fig. 3:**
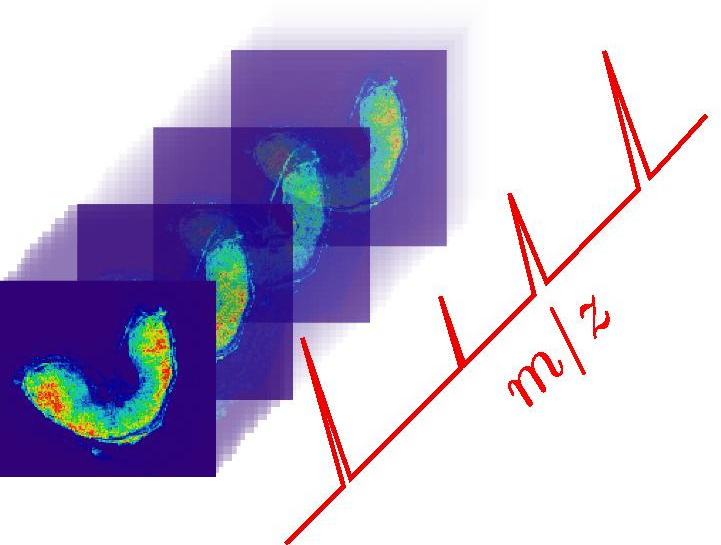
MALDI–MS image: 3D datacube, or hyperspectral image. A spectrum (in red) is associated to each pixel and describes the molecular content at this position. Each molecule is associated to an ion image, and is characterized by its mass-to-charge ratio (*m/z*) and relative ion intensity

**Fig. 4:**
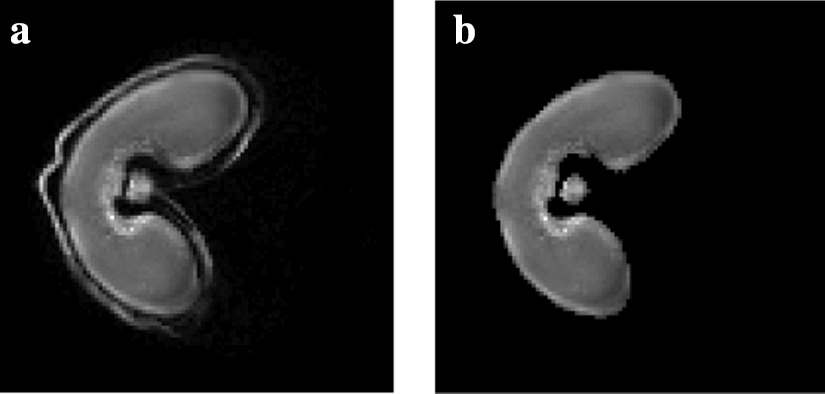
Segmentation of MRI images. **a** Original MRI image and **b** resulting segmented image. The pericarp (outer structure) of the wheat grain is removed in the segmented grain so as to compare MRI and MALDI–MS images more easily

**Fig. 5:**
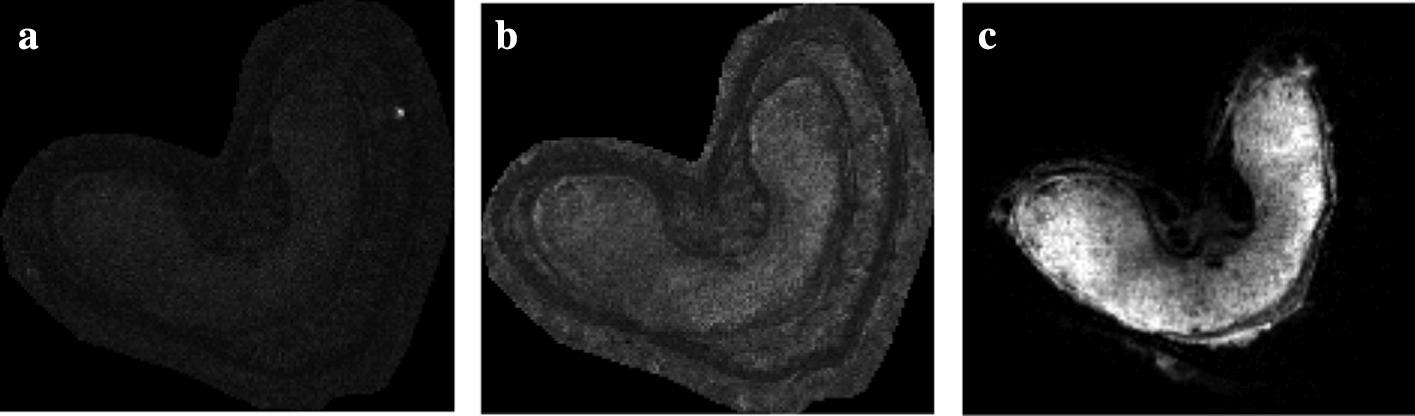
Examples of MALDI–MS images exhibiting differences in intensities for different mass-to-charge ratios. **a** 432.92, **b** 476.28 and **c** 611.33. **a** Image resulting from an artefact: high signal detected outside of the sample. **b** Noisy image. **c** Spatially coherent image

**Fig. 6:**
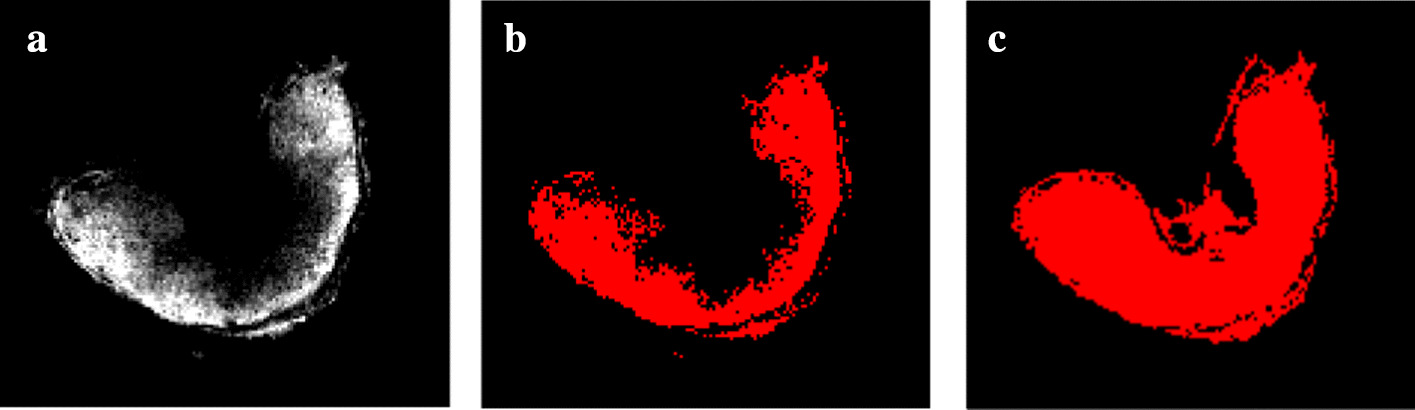
Segmentation method for MS images. Refinement of a segmented shape by region growing on a subset of relevant ion images. **a** Initial ion image and **b** associated segmented shape obtained by region growing. **c** Complete segmentation obtained by applying the region growing procedure on the full subset of images

The same wheat grains are imaged in MALDI–MSI. The sample preparation is described in Fanuel et al. [13]; first, the tissue is sectioned in several transverse slices, then the tissue is digested by specific enzymes, and the MALDI–MS matrix is sprayed onto the sample. Images are acquired using a rapiFlex TissueTyper MALDI-MS spectrometer (Bruker, Daltonics, Bremen). Molecule ionization is done by a 355 nm laser operating at 10 kHz (BIA-BIBS platform). The resulting MALDI–MS image is an hyperspectral image, that is to say a three dimensional datacube with two spatial dimensions (image height and width) and a spectral dimension (see Fig. 3). Molecules on the spectral dimension are characterized by their mass-to-charge (*m/z*) ratio. Each pixel corresponds to a spectrum, and encloses the molecular distribution at this position. The image size is $152\times 138\times 65000$ pixels (width, height, number of points in a spectrum). The pixel size is 25 $\upmu \mathrm{m}$, and the spectral resolution is 0.017. The space between consecutive slices is 80 $\upmu \mathrm{m}$.

The steps of the proposed workflow are detailed in the following sections, with an emphasis on the new proposed methods and their efficiency.

### Pre-processing of MRI images

The MRI images are denoised using a non-local means method specifically suited for the removal of Rician noise [14]. This method replaces a pixel value by the average intensity in the image, weighted by the intensity similarity to the target pixel.

The 3D density image, that is to say the image at time $t=0$, is estimated by fitting a mono-exponential function on the signal intensities. This function is adjusted by non-negative least squares regression, with the Levenberg–Marquardt algorithm.

The intensities between the grain and the background are clearly separated. Thus, a simple thresholding scheme is used for the segmentation of the wheat grain. The outer structure of the wheat grain, called pericarp, is not visible on MS images. It is removed from the segmented image by applying a morphological opening for the subsequent registration step (see Fig. 4), with the radius of the structuring element set to 2 pixels [15].

Finally, a 2D transverse slice (*x*–*y* plane) is chosen in the 3D density image to match the 2D MALDI–MS image. It is chosen as the MRI slice that is the closest to the analyzed MALDI–MS slice along the *z*-axis, and is found by utilizing the resolution and the slice thickness of both images.

### Pre-processing of MALDI–MS images

The spectral dimension in the MALDI–MS image is reduced using peak detection and peak alignment.

*Spectra processing* MALDI–MS images mainly contain non relevant information, due to the large amount of points associated with noise in the spectral dimension. Peak detection consists in identifying local maxima in the spectra whose intensity is above the signal noise. Ideally, the peak detection methods must be complete, that is to say they identify all true peaks across all spectra; specific, that is to say they contain no false positives; and efficient. Yang et al. [16] compare existing methods for peak detection in MS images, and conclude the continuous wavelet transformation achieves the best trade-off between completeness and specificity. However, the computation time is prohibitive as the number of points increases.

We propose a complete and efficient peak detection method, based on the *prominence* of the peaks relative to the signal noise [17]. The prominence of a peak is defined as the height of this peak relative to the height of the neighbouring peaks. This measure discards local maxima which come from irregularities in the signal. Let *y*(*x*) be a spectrum, *P* the set of local maxima *y*, $p = (x_p, y_p) \in P$ a peak, *w* the half-length of a window, and $m_p = (x_m, y_m) \in P$ the peak such that $m_p$ is the closest peak to *p* with $y_m \ge y_p$ for $x \in [-w, w]$. If $x_m \ne x_p$, the prominence of *p* is the vertical distance between *p* and the local minimum between *p* and $m_p$, else the prominence is equal to $y_p$.

We define the *local prominence* as the ratio between the prominence and the estimated local noise in the signal. This local measure is specifically suited to peak detection for varying peak intensities across the *m/z*-axis. In practice, all spectra are considered individually. The local noise is estimated as the median of absolute deviations in a window. First, local maxima are extracted and constitute an initial set of peaks. Then, this set is refined by selecting peaks whose local prominence values are above a given noise threshold.

Small *m/z* variations are observed for the same molecule at different pixel locations, due to instrumental instabilities and sample preparation imprecisions (irregularities in the tissue flatness). Peak alignment consists in mapping the previously detected peaks to a common *m/z* value, in order to facilitate spectrum comparison. Here, peaks are aligned by matching peaks to detected peaks in the mean spectrum [18].

*Segmentation* After peak detection and alignment, selected ion images do not necessarily reflect the shape of the embedded object (see Fig.  5). Moreover, ion images might highlight different parts of the wheat grain. Thus, a complete segmentation of the wheat grain is obtained by region growing on a subset of relevant images, that is to say images where parts of the wheat grain are apparent.

First, we extract a subset of relevant ion images. Alexandrov and Bartels [19] introduce a new measure called spatial chaos, which quantifies abrupt intensity variations in an image. This measure characterizes how points with high intensity values are spread, using various binarized versions of the image. The spatial chaos values are generally low for relevant ion images, and high for noisy images. This does not hold for noisy images with intensity artefacts (see Fig. 5a), and when the variance noise is low (see Fig. 5b).

Our method is adapted to discard these images and builds on the approach proposed by Alexandrov and Bartels [19]. We define a new measure, called spatial coherence. Our measure considers the area of the largest connected component in different binarized versions of the image. In the following, the intensities of each ion image are normalized between 0 and 255. Let *I* be an ion image, *t* a threshold, *T* a set of thresholds, $B_t(I)$ a binarized version of *I* obtained with *t*, *C*(*B*) the set of connected components in *B*, then the spatial coherence *S*(*I*) is defined as :

$$\begin{aligned} S(I) = \min _{t \in T}~\max _{c \in C(B_t(I))} |~c~| \end{aligned}$$

In practice, we choose *T* as a set of thresholds defined by the following quantiles of intensity values : [0.6, 0.7, 0.8, 0.9]. The spatial coherence values are low for noisy images, and high for relevant images. A subset of relevant ion images is obtained by selecting images whose spatial coherence values are above a given threshold.

Second, a region growing procedure is applied on the subset of relevant ion images. The initial seed point is chosen as the point with the highest intensity in the subset. An initial segmented shape is obtained by applying the region growing procedure on the ion image containing the seed point. This segmented shape is refined incrementally by iterating over each ion image, and ultimately captures the shape of the wheat grain (see Fig. 6). The pixel intensities in the segmented shape correspond to the average intensities in the subset of relevant images.

### Registration

At this stage, both segmented shapes in MALDI–MS and MRI images can be matched. Registration methods aim at finding the transformation which best aligns two objects. The transformation parameters are estimated by optimizing a metric which quantifies the similarity between both images. This metric can be based on intensity values, or the geometrical shape of the objects. In the following, we are interested in automatic methods to register the segmented wheat grain in MALDI–MSI with respect to its counterpart in MRI. First, we globally align the objects with a linear registration method using an affine transform. Then, we use a deformable registration method to compensate for the local geometrical deformations induced by sample preparation in MALDI–MSI (see “Image acquisition” section). We choose the MR image as the reference image because it encloses the non-deformed shape. The resulting deformed MS image is thus easier to interpret biologically because molecules are distributed across anatomical regions of the tissue.

Regarding the linear registration method, the affine transformation involves translation, rotation and scaling and is initialized by aligning both centers of the objects using moments. Both images have a bimodal intensity distribution. Thus, we choose the mutual information for the similarity metric, which is a measure estimating the statistical dependence between the intensity distributions of both images. This metric is optimized by a regular gradient descent optimization algorithm.

The quality of a deformable registration method is determined by the shape resemblance and the intensity fidelity between the two images, after registration. Deformable registration methods can be classified into different categories depending on the parametrization of the model [20]. Parametric methods, such as B-spline free form deformations (FFD), involve a transformation with a small number of parameters. However, it is difficult to choose an adequate number of parameters to obtain a good compromise between shape resemblance and intensity fidelity. On the other hand, non-parametric methods, often referred to as variational methods, map each pixel in the image to a displacement vector. Modersitzki [21] describes variational methods as the minimization of a functional involving two terms: (a) external forces: the similarity metric, and (b) internal forces: the regularization term. The minimization of the similarity metric brings similar pixels close to one another, whereas that of the regularization term avoids local irregularities in the vector field. This is particularly important in regions where the intensity difference between both images is null or low. We choose the sum of squared differences as the metric because both images of the wheat grain have similar intensities. In a more traditional multimodal case, the mutual information metric is more suited because intensities do not necessarily match. Moreover, we choose the elastic regularization strategy because its parameters $\mu$ and $\lambda$ grant a fine control over the rigidity of the material, which is necessary to preserve the internal shape of the object [22].

Finally, the estimated affine and deformable transformations are applied to the MALDI–MSI datacube, that is to say to each individual ion image. For both registration steps, the intensities are obtained by nearest-neighbor interpolation, so as to not create extraneous intensities in the ion images.

### Joint statistical analysis

We aim at finding the molecules in MALDI–MSI ion images whose distribution correlates with the water distribution in MRI, and identify groups of molecules who share the same distribution pattern. Ovchinnikova et al. [23] evaluated several measures to identify spatial correlations between pairs of images. For measures requiring no machine learning, the cosine distance yielded the closest results to those obtained by experts. However, the complexity is quadratic when searching for groups of similar ion images.

We opt for a matrix-factorization approach. Statistical analysis is achieved in three steps: (a) matrix factorization of the MALDI–MS image, (b) projection of the density MRI image in the reduced space produced by matrix factorization and (c) selection of the MALDI–MS ion images which are closest to the MRI image in this space.

The intensities of each indivual ion image are normalized on a 0–255 range. The MALDI–MSI datacube is reshaped into a two-dimensional matrix where the rows correspond to pixels and the columns to mass-to-charge ratios, that is to say molecules. Siy et al. [24] evaluate several matrix factorization techniques in the context of MALDI–MS images : Principal Component Analysis (PCA), Non-Negative Matrix Factorization (NMF), Independent Component Analysis (ICA). These methods produce two smaller matrices with a fixed number of components and whose product is an approximation of the original matrix. One matrix encloses component images, that is to say the contribution of each pixel in the components, and the second matrix corresponds to the contribution of molecules in the components. Both ICA and NMF produce component images with less noise than PCA. We choose NMF because its non-negativity constraint makes it possible to interpret the component images more easily.

## Results

In this section, the efficiency of our workflow is shown by comparison to state-of-the-art methods, using wheat grain images as an example. Additional information about input files, parameters, and result reproducibility is available in Additional file 1.

### Peak detection

Our peak detection algorithm is compared to the method based on continuous wavelet transformation (CWT), which Yang et al. [16] found to give the most complete results, with the lowest amount of false positive peaks. Both methods are compared using synthetic simulated data and real data. The synthetic simulated dataset is a MALDI–MS image containing a hundred spectra, where theoretical peaks are known [25]. The real dataset is a subset of a hundred pixels of the MALDI–MS image of the wheat grain. The spectra were annotated manually and produce a subset of theoretical peaks.

For both CWT and our algorithm, the parameters were set such that the totality of theoretical peaks were identified with as few false positive peaks as possible. The efficiency of our method is assessed by two metrics: (a) the completion time *t* per spectrum, in milliseconds (b) the precision *p*, i.e. the ratio between the number of theoretical peaks with respect to the number of detected peaks.

The results are presented in Table 1. Our method has lower precision values (9.68% and 11.2% vs. 13.0% and 14.8% for the synthetic and real dataset, respectively), which means our method yields slightly more false positive results. In practice, 18 supplementary peaks are detected on average by our method. This is negligible with regard to the average number of detected peaks ($n=600$). The computation time per spectrum for our method is much lower, which makes it the best candidate for large MSI datasets.

**Table 1:** Precision *p* and computation time *t* per spectrum (in milliseconds) for peaks detected on synthetic and real data, using our algorithms (Ours) and continuous wavelet transformation (CWT)

		p	t (ms)
Synthetic	CWT	**0**.**130**	1200
	Ours	0.0968	**19**
Real	CWT	**0**.**148**	8600
	Ours	0.112	**47**

The best values for each parameter appear in bold

CWT is not suited to large MALDI–MS images because of its computation time

### Segmentation

Our segmentation method is assessed by analyzing the images extracted in the relevant set, and quantifying how much each image in the relevant set adds to the final segmentation.

The segmented objects are compared by estimating the correlation between the curvature distributions of the object contours. The curvature is estimated locally at each point of the object contour using the Voronoi Covariance Measure (VCM, Cuel et al. [26]). The VCM is a covariance measure of Voronoi cells located around the target point. This measure is linked to the curvature, since Voronoi cells are restricted in areas with extreme curvature values, and are elongated in flat areas. The curvature distributions of the MSI and MRI images are compared by computing the Spearman correlation coefficient.

Our method is compared to the spatial chaos method (see “Pre-processing of MALDI–MS images” section). For both methods, we assess whether the shapes in the segmented MS and MR images are similar. We compare the distributions of curvature values on the shape boundary between the MR image and the segmented MS image. First, the curvature values are estimated using the Voronoi covariance measure (VCM) estimator [26]. Then, the curvature distributions are compared using the Spearman correlation coefficient [27], which measures the strength of a monotonic relationship between two distributions. The threshold on either the spatial coherence or the spatial chaos measures are chosen such that the Spearman correlation coefficient is the highest, that is to say the shapes in the segmented MS and MR images are most similar.

**Fig. 7:**
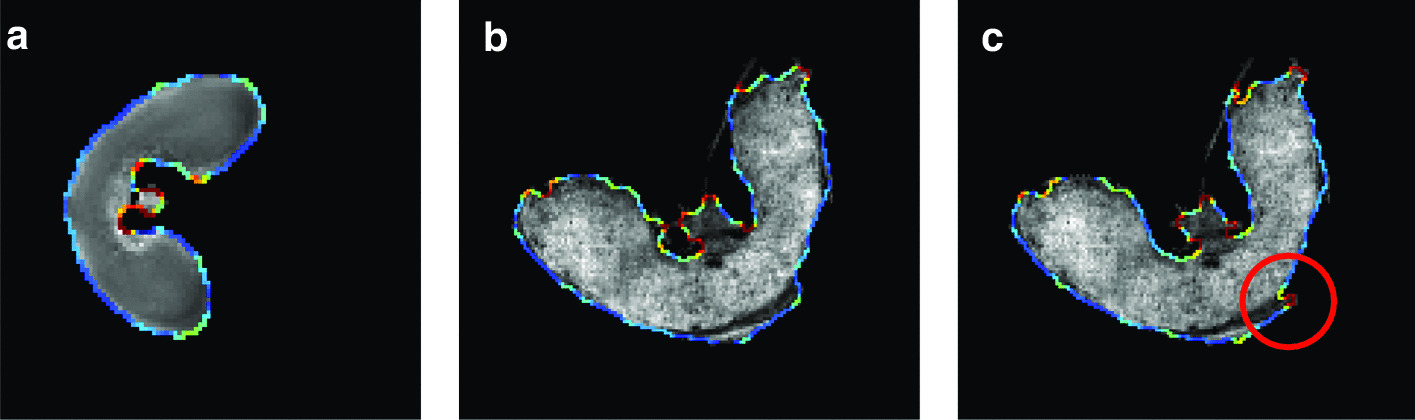
Validation of the segmentation approach. Curvature values are computed on the contours of the **a** MR image, and MALDI–MS segmented images using **b** our method and **c** the spatial chaos measure. The values range from low (flat areas, in blue) to high (salient points, in red). The values are close in all images, but the spatial chaos measure yields high curvature values in the posterior part of the tissue (circled in red) which are low for the MR image. This reflects a slightly incomplete segmentation in the case of the spatial chaos measure

**Fig. 8:**
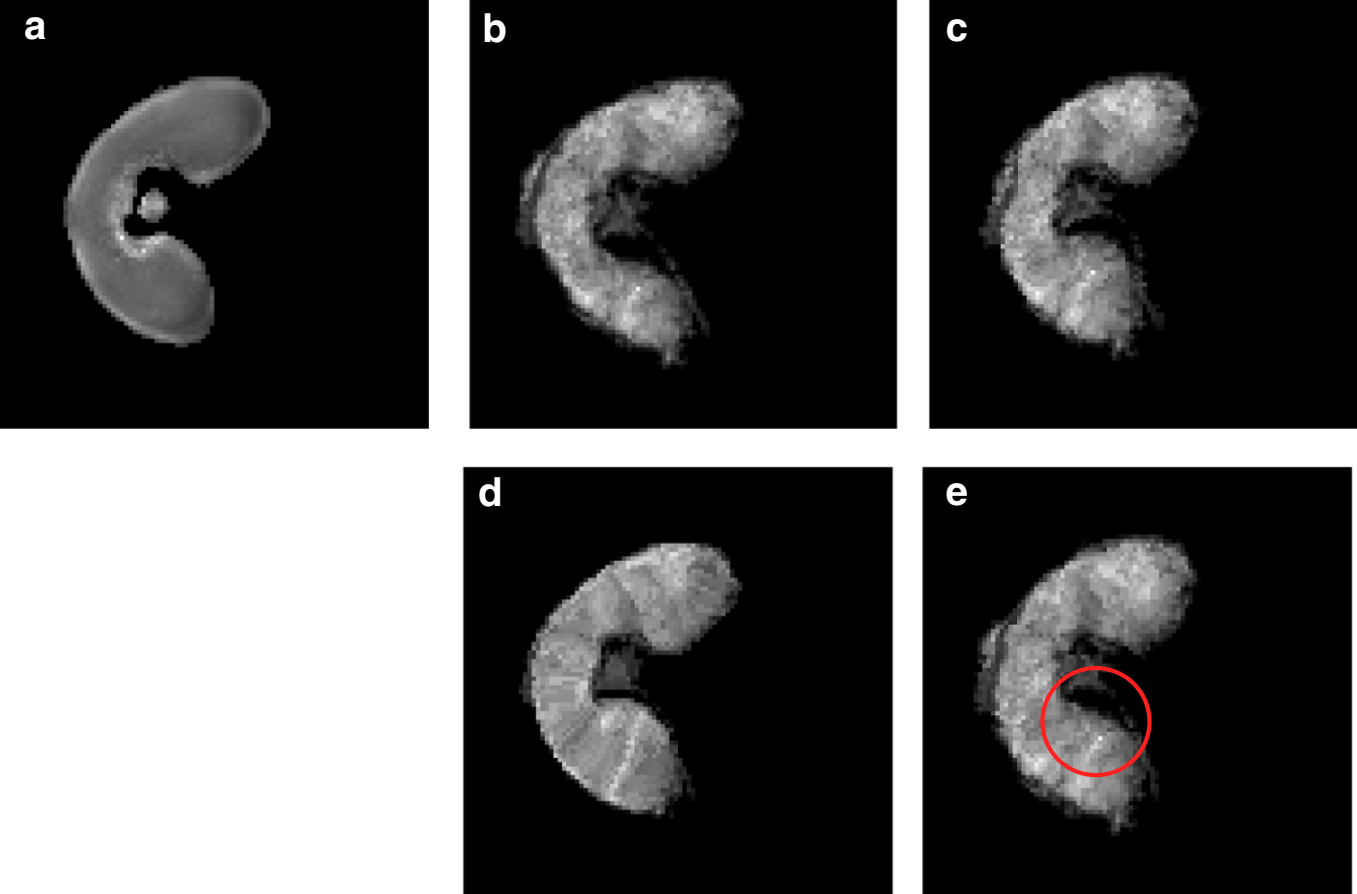
Registration of the MALDI–MSI image onto the MRI image. Registration of the segmented MALDI–MS image onto the **a** denoised MRI image: **b** linear registration followed by **c** the variational method of Modersitzki [21] or **d**, **e** free form deformation models with two different parameters ($\text {FFD}_1$ and $\text {FFD}_2$). **e** The circled area (in red) shows a difference in shape compared to the MRI image

The curvature distributions follow the same trend (see Fig. 7). The Spearman correlation coefficient [27] for the final segmentation is 0.540 with our measure, and 0.438 for the spatial chaos. This discrepancy is due to the number of ion images found and used by each method: 31 images for our measure versus 8 images for the spatial chaos measure. The spatial chaos approach misses several relevant ion images, which results in an incomplete segmented image. Thus, our method provides a segmentation which is more precise.

### Registration

The registration approach is evaluated by quantifying the similarity between the pixel intensities of the original and the registered MALDI–MS image. This is achieved by computing the mutual information $m_I$. Moreover, various metrics are used to measure the shape resemblance between the MRI and registered images. The segmented images are binarized, and three metrics are used: (a) the precision *p*, i.e. the ratio between the number of common pixels and the number of pixels in the MALDI–MS image, (b) the recall *r*, i.e. the ratio between the number of common pixels and the number of pixels in the MRI image and (c) the F-measure $F = 2 \cdot \frac{p \times r}{p+r}$.

The registration method was evaluated by comparison with a free form deformation model (FFD) consisting in a grid of B-spline control points. Two sets of parameters were used to obtain the best *F* values ($\text {FFD}_1$) on one hand, and the best $m_I$ values ($\text {FFD}_2$) on the other hand.

**Table 2:** Registration metrics for the evaluation of the used method (affine + variational) by comparison to the free form deformation model with two sets of parameters (FFD_1_ and FFD_2_): precision *p*, recall *r*, *F*-measure, and mutual information *m*_*i*_. The variational method offers the best compromise between intensity fidelity and shape similarity

	*p*	*r*	*F*	$m_I$
Affine	0.876	0.890	0.883	0.994
Affine + Variational	0.951	0.975	0.963	0.454
Affine + $\text {FFD}_1$	0.940	0.984	0.961	0.419
Affine + $\text {FFD}_2$	0.871	0.958	0.912	0.471

**Fig. 9:**
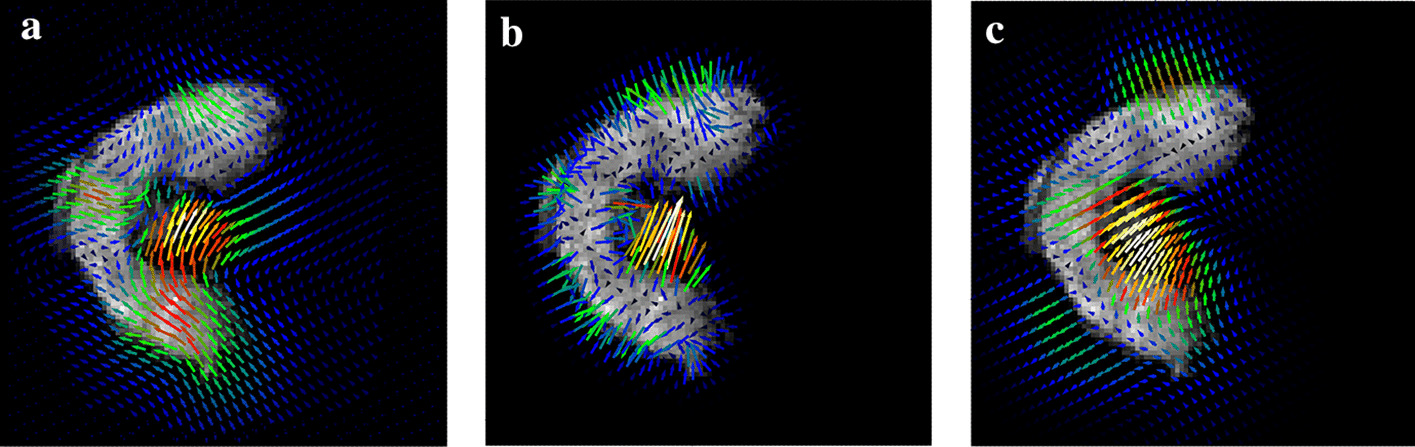
Vector fields obtained by deformable registration methods. The vectors correspond to the displacements applied to the MS image to match the MR image, obtained by : **a** the variational method of Modersitzki [21] or free form deformation models with two different parameters **b** $\text {FFD}_1$, **c** $\text {FFD}_2$. The vector field resulting from the variational method is more homogeneous than that of $\text {FFD}_1$ and more precise than that of $\text {FFD}_2$

**Fig. 10:**
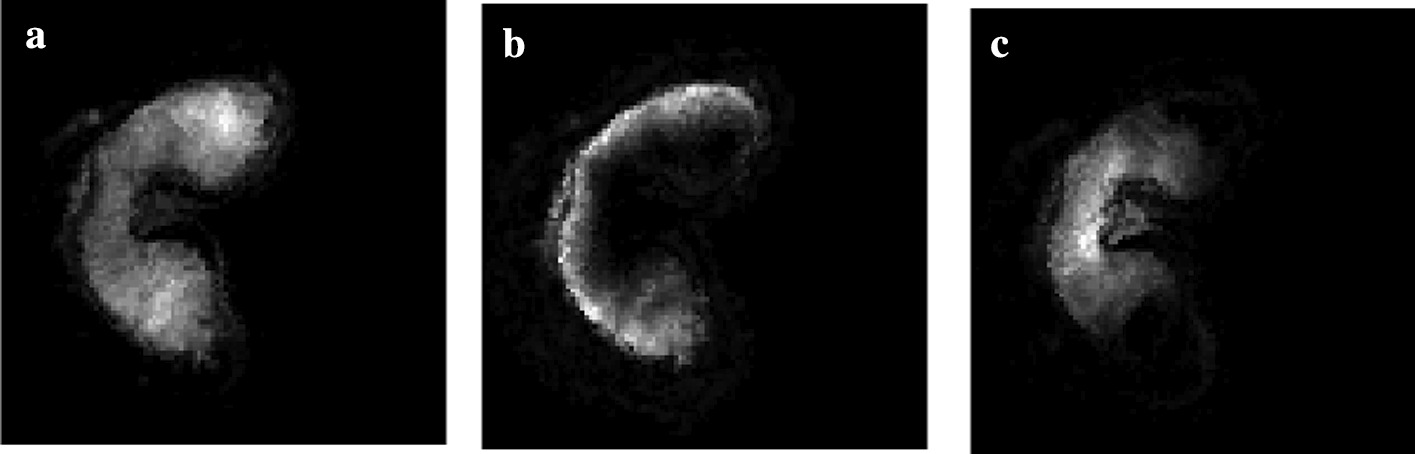
Component images and molecule distribution. Selected component images resulting from NMF, exhibiting different molecule distributions across the wheat grain: **a** lobes, **b** outer layers, **c** transfer cells

The results are presented in Table 2 and Fig. 8. Our method gives the best F-measure (0.963) by comparison to the FFD method. The intensities before and after registration are close, with a mutual information value of 0.454 bits. By contrast, the registered image with $\text {FFD}_1$ is less similar in shape (96.1%), and very different in terms of intensities (see Fig. 8d). The registered image with $\text {FFD}_2$ is more faithful in terms of intensities, but the shape is not as precise (see Fig. 8e). Indeed, the deformation of the variational method is more homogeneous than the one obtained with $\text {FFD}_1$, and more precise in terms of vector orientations than the one obtained with $\text {FFD}_2$ (see Fig. 9). The selected method offers the best compromise between intensity fidelity and shape resemblance.

### Joint statistical analysis

We choose the number of components for NMF such that the coefficient of determination $R^2$ is greater than 0.95. Spatial correlations were evaluated by comparison to those found by the cosine distance, which was shown to be the most precise measure (see “Joint statistical analysis” section). Each ion image was ranked according to its similarity to the MRI image. On average, ion rankings deviate from the rankings obtained by the cosine distance by 2.9%. Thus, our method provides similar results.

NMF yields several component images which highlight specific regions in the wheat grain (see Fig. 10). The contribution matrix showed ions assigned to arabinoxylans (i.e. polysaccharides) with low degrees of polymerization were located in the grain lobes (see Fig. 10a), feruloylated arabinoxylans (i.e. arabinoxylans with ferulic acid residues) were in the posterior outer layers of the wheat grain (see Fig. 10b), and arabinoxylans with various degrees of polymerization and without chemical modification were observed in the center of the grain (see Fig. 10c). The distribution of feruloylated arabinoxylans in posterior areas of the grain was previously observed in the literature [28].

**Fig. 11:**
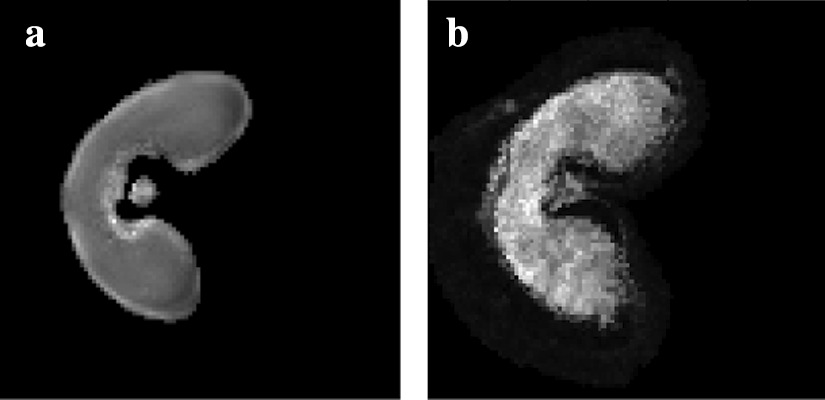
Projection of the MRI image in the NMF reduced space. Difference between **a** the original MRI image and the **b** MRI image projected in the NMF space and obtained by linear combination of the component images. Both images have similar intensities. The NMF does not result in major information loss

**Fig. 12:**
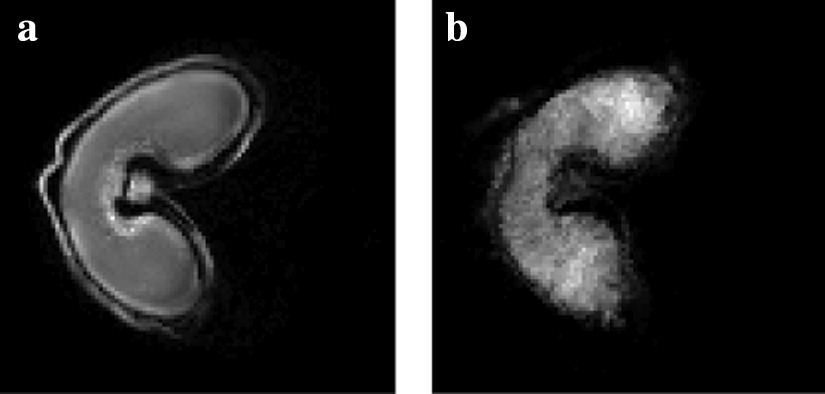
Example of strong spatial correlations between MRI and MSI. Example of a close spatial correlation between **a** the MRI image and **b** the MALDI–MS ion image of *m/z* 785.30, corresponding to an arabinoxylan with a degree of polymerization of 5 with two acetyl groups

Spatial correlations are established with the projection of the MRI image onto the reduced space produced by NMF. The projected image is formed by a linear combination of the NMF component images. We aim at quantifying the amount of lost information induced by the projection. The average absolute difference in intensities between the original MRI image and the projected image is only about 4.2% (see Fig. 11). Thus, the projection in the NMF space preserves the intensities of the original image.

Both these results support the relevance of the selected matrix factorization approach.

The molecules which correlate the most with the distribution of water are arabinoxylans with low degrees of polymerization and an acetyl group. These molecules are distributed mainly in the lobes of the grain (see Fig. 12). We suspect that the acetylation of arabinoxylans renders the cell wall network better able to uptake water by changing the hydrophobicity properties of the polysaccharides, as hinted in Gille and Pauly [29]. Thus, the joint analysis of MSI and MRI images identified acetylated arabinoxylans as new candidates likely to contribute to the absorption of water in the developing grain.

## Discussion

### Applicability

The proposed workflow is particularly suited for exploratory research. The present study allows for the discovery of new candidates involved in the uptake and distribution of water in the wheat grain. The workflow does not depend on the case study and can be applied on any dataset which satisfy two main conditions. First, our segmentation approach relies on the hypothesis that the object has the same overall shape, or contain similar features in both images. Second, our registration and statistical analysis methods make the assumption that the intensity distribution is comparable between MRI images and at least one ion image in MSI. These broad conditions make our workflow applicable to a large number of MSI and MRI images.

Our workflow can be reused in combination with other modalities. MSI and fluorescence microscopy can be merged in order to discover molecular partners of fluorescent proteins, such as the study led in Jones et al. [30]. On another note, MSI and Raman spectroscopy images can be analyzed jointly to identify specific regions in the sample [31].

### Research prospects

Registration methods aim at matching two ore more images by finding correspondences between images. However, correspondences do not always exist in specific areas of the images. For instance, the signal might be extremely low in certains areas of the tissue in MS images. In our case, the pericarp of the wheat grain is apparent in MRI images but absent from MS images. We alleviated this problem by discarding the pericarp during the segmentation of the MRI image. We are currently working on finding missing correspondences automatically during the registration. This can be done by building probabilistic correspondence maps [32] or using geometrical constraints [33].

Our workflow processes 2D MALDI–MS images. In the future, several images will be imaged, providing a 3D representation of the molecule distribution across the sample. Three-dimensional extensions of our methods need to be considered. Regarding the segmentation procedure, the spatial coherence measure is independent of the dimensionality, and a 3D region growing procedure can be used. The variational registration method of Modersitzki [21] is suited to 3D images. In order to perform the joint statistical analysis in 3D, multiblock methods , such as multiple co-inertia analysis [34], can be used.

## Conclusions

In this article, we have proposed a new workflow for the fusion of MRI and MSI images which involves precise and efficient methods. There are two main challenges using MALDI–MS images in an image fusion task. First, these images enclose a large amount of data. Secondly, tissue preparation induces local sample deformations. The selected and proposed methods are specifically suited to address these problems. In particular, we proposed a new peak detection method which achieves fast computation while being complete. Our new segmentation approach utilizes the information contained in numerous ion images, which provides a complete and precise segmented MALDI–MS image. The selected registration approach compensates for local irregularities. Finally, the spatial correlations are established by a dimension reduction technique which limits data loss. We validated each step of our workflow by a quantitative evaluation involving comparison with state-of-the-art methods. Our workflow provides accurate results across all steps, which demonstrates its relevance in an image fusion task involving MSI.

## Supplementary Information

**Additional file 1.** User manual, and Parameter setting file. This file describes the parameters used for each algorithm, in order to reproduce the results.

## Data Availability

The synthetic dataset used for peak selection is available at : https://bioinformatics.mdanderson.org/public-datasets/. The other datasets used and analysed during the current study are available from the corresponding author on reasonable request. The source code and documentation with examples are available online at https://github.com/fgrelard/Esmraldi. Please refer to the “User guide—Parameter setting” additional file online for a practical guide on the usage of the workflow and a description of the algorithm parameters. Our results can be reproduced by running the compute capsule available here : https://codeocean.com/capsule/9536349/tree.

## Abbreviations

CWT: Continuous wavelet transform
FFD: Free-form deformation model
MALDI: Matrix assisted laser desorption ionization
MRI: Magnetic resonance imaging
MSI: Mass spectrometry imaging
NMF: Non-negative matrix factorization
VCM: Voronoi covariance measure
