# Supplementary material for: Esmraldi: efficient methods for the fusion of mass spectrometry and magnetic resonance images
Source: BMC Bioinformatics. 2021 Feb 8;22:56. doi: 10.1186/s12859-020-03954-z (PMC7869484; doi:10.1186/s12859-020-03954-z)
Supplement: Supplementary file 1 — Additional file 1. User manual, and Parameter setting file. This file describes the parameters used for each algorithm, in order to reproduce the results. [file 12859_2020_3954_MOESM1_ESM.pdf]

A new workflow for the fusion of Mass Spectrometry and Magnetic Resonance images

User manual – Parameter setting

Grélard et al.

The following document is aimed at users of the workflow. We explicitly describe the parameters used for each step and their impact on the results.

1 Data format

MS images must be in the imzML format. Various tools are available online to convert from proprietary format to mzML or imzML (e.g. imzMLConverter).

Complementary images can be in any ITK format (.png, .tif, .hdr. . .).

2 Parameter setting

2.1 Spectra processing

First, the spectra are normalized by their total ion count, that is to say the sum of the intensities of each spectrum.

Then, the peaks in the wheat grain MS image are selected according to the local prominence measure. Peaks above a local prominence value of 75 are retained. The chosen threshold defines the number of peaks: the lower the threshold, the more complete the results, but the higher the false positive rate.

Regarding the alignment procedure, peaks are aligned to a peak in the mean spectrum if they are within 0.2 of this peak's mass-to-charge ratio.

2.2 Segmentation

Regarding the segmentation of the MR image, we denoise the image using the non-local means method of Wiest-Daesslé et al. (2008). This method aims at restoring noisy pixel intensities by considering patches, that is to say neighboorhoods in the image, which have similar intensity distribution. Here, the patch size is 5x5, the search radius for similar patches is restricted to 6, and two patches are considered similar if the absolute difference between the mean intensities of the patches is less than 1.75 times the noise spread.

Regarding the segmentation of the MS image, we use the spatial coherence measure to obtain a set of non-noisy ion images. The ion images which have a spatial coherence measure above a given threshold are kept. Intuitively, this threshold should be chosen as the minimum area of regions of interest in the image. In our case, we set the threshold at 2100.

We validate our approach by comparison to the spatial chaos measure. This measure reflects the average distance between pixels in an image, and will be high for noisy images. In our case, we select a threshold of 1.003.

For both approaches, the measures are obtained on the set of quantile thresholds $\{60, 70, 80, 90\}$.

Our validation approach consists in comparing the curvature distributions on the boundary of the segmented MR and MS images. First, the segmented images are binarized and the boundary is extracted. The boundary is converted to a closed digital curve with 4-connectivity. We use the Voronoi Covariance Measure (Cuel et al., 2014) to estimate the curvature at each point of the curve. This method integrates the shape of Voronoi cells locally. In our case, the search radius r for the Voronoi cells is set to 4, and the bounding radius R for Voronoi cells is set to 5. Finally, the curvature distributions are compared by retaining the best Spearman correlation coefficient over the set of MS curvature distributions shifted by a factor $\in [0, n]$ where n is the number of points on the boundary of the MS image.

2.3 Registration

The affine registration is done by using the similarity transform. We use Mattes' mutual information, with a number of bins equal to 27 such that, on average, each bin contains 10 points. Optimization is done by the regular step gradient descent algorithm, with a learning rate of 1.1 and a relaxation factor of 0.8. The learning rate is the quantity by which the gradient is followed at each step. A large value (> 1) for the learning rate generally allows for faster convergence but might result in "missed" minima. Moreover, when the direction of the gradient changes, the learning rate is reduced by the relaxation factor. Large values (> 0.5) are used when the images to register have large differences in orientation or scale.

Regarding the variational approach, we use the sum of squared differences because the intensities are close between both images. In a more traditional multimodal case, the mutual information metric is more suited. We choose the elastic regularization strategy because its parameters μ and λ grant a fine control over the rigidity of the material, which is necessary to preserve the internal shape of the object. When both μ and λ are close to one, the displacement field is constrained to be homogeneous; values close to zero mean the displacement field is more accurate from the metric perspective but also more inconsistent. Here, we set $\mu = 0.8$ and $\lambda = 0.9$. Finally, the gradient descent step is set to 7.5×10^{-4} with 5 multi-resolution levels.

The free form deformation models use a grid of B-Spline control points. For both cases FFD_1 and FFD_2, the spline order is 2, but the number of control points is different : 25x25 for FFD_1 and 9x9 for FFD_2. The metric used is the normalized cross-correlation metric. A quasi-Newton optimizer (LBFGS) is used with a step length of 1.5 and search line accuracy of 1.2.

For all registration steps, the nearest-neighbor interpolation scheme is used.

2.4 Joint statistical analysis

The joint statistical analysis is performed by Non-Negative Matrix Factorization (NMF). The matrices are initialized by a dense variant of the Nonnegative Double Singular Value Decomposition (NNDSVDa), which leads to faster convergence and lower error rate (Boutsidis and Gallopoulos, 2008). Matrices are obtained by the multiplicative update rule. The number of components is set to 7, that is to say the lowest number of components such that the reconstruction error is greater than 0.95.

Author details

References

Boutsidis, C., Gallopoulos, E.: SVD based initialization: A head start for nonnegative matrix factorization. Pattern Recognition **41**(4), 1350–1362 (2008). doi:10.1016/j.patcog.2007.09.010

Cuel, L., Lachaud, J.-O., Thibert, B.: Voronoi-based geometry estimator for 3d digital surfaces. In: Advanced Information Systems Engineering, pp. 134–149. Springer, Heidelberg (2014). doi:10.1007/978-3-319-09955-2_12. https://doi.org/10.1007/978-3-319-09955-2_12

Wiest-Daesslé, N., Prima, S., Coupé, P., Morrissey, S.P., Barillot, C.: Rician Noise Removal by Non-Local Means Filtering for Low Signal-to-Noise Ratio MRI: Applications to DT-MRI. In: MICCAI 2008 vol. 5242, pp. 171–179. Springer, Heidelberg (2008). doi:10.1007/978-3-540-85990-1_21. http://link.springer.com/10.1007/978-3-540-85990-1_21 Accessed 2019-05-10
